# Decreased Peripheral Blood *ALKBH5* Correlates with Markers of Autoimmune Response in Systemic Lupus Erythematosus

**DOI:** 10.1155/2020/8193895

**Published:** 2020-06-25

**Authors:** Qing Luo, Biqi Fu, Lu Zhang, Yang Guo, Zikun Huang, Junming Li

**Affiliations:** ^1^Department of Clinical Laboratory, The First Affiliated Hospital of Nanchang University, Nanchang, Jiangxi 330006, China; ^2^Department of Rheumatology, The First Affiliated Hospital of Nanchang University, Nanchang, Jiangxi, China

## Abstract

Although it has been proved that the epigenetic modification of DNA and histones is involved in the pathogenesis of systemic lupus erythematosus (SLE), there is no study to explore whether the modification of N6-methyladenosine (m6A) in RNA is involved. In this study, the mRNA levels of m6A “writers” (*METTL3*, *MTEEL14*, and *WTAP*), “erasers” (*FTO* and *ALKBH5*), and “readers” (*YTHDF2*) in peripheral blood were determined by quantitative reverse transcription-polymerase chain reaction (qRT-PCR). The results demonstrated that the mRNA levels of *METTL3*, *WTAP*, *FTO*, *ALKBH5*, and *YTHDF2* in peripheral blood from SLE patients were significantly decreased. The levels of *ALKBH5* mRNA in SLE patients were associated with anti-dsDNA, antinucleosome, rash, and ulceration. Multivariate logistic regression analysis showed that the level of ALKBH5 mRNA in peripheral blood is a risk factor of SLE (*P* < 0.001). Moreover, our results suggested that there was a positive correlation between m6A“writers” (*METTL3* and *WTAP*), “erasers” (*FTO* and *ALKBH5*), and “readers” (*YTHDF2*) in SLE patients. This study suggests that the mRNA level of *ALKBH5* in peripheral blood may be involved in the pathogenesis of SLE.

## 1. Introduction

The systemic lupus erythematosus (SLE) is a chronic and incurable autoimmune disease characterized by intermittent episodes of increased disease activity that require treatment with immunosuppressive agents [1, 2]. Although there have been many studies trying to elucidate the pathogenesis of SLE, so far it has not been fully elucidated. Existing studies have demonstrated that the dysfunction of immune cells such as T cells, B cells, monocytes, neutrophils, and dendritic cells plays important roles in the pathogenesis of SLE [3–6]. Further elucidation of the aetiology of SLE is of great significance for the development of possible targeted and individualized therapy for SLE [7].

In recent years, epigenetic modifications have been demonstrated to play an important role in the genesis and development of SLE [8, 9]. N6-methyladenosine (m6A) modification is the most prevalent and evolutionarily conserved modification which occurs in nearly all types of RNAs and in most organisms [10]. This modification can be installed by adenosine methyltransferases, reversed by demethylases, and recognized by some RNA-binding proteins [11]. M6A methyltransferase complex, known as the m6A “writer” complex, contains methyltransferase-like 3 (METTL3), methyltransferase-like 14 (METTL14), and Wilms tumor 1-associating protein (WTAP), which functions by depositing the m6As in mammalian mRNA [12]. Fat mass and obesity-associated protein (FTO) and a-ketoglutarate-dependent dioxygenase alkB homolog 5 (ALKBH5) are selective demethylases capable of regulating gene expression and cell fate through oxidative removal of the methyl group in m6A-containing substrates, usually considered as m6A “erasers” [13]. Some RNA-binding proteins such as YT521-B homology domains 2 (YTHDF2) which can recognize m6A modification, decode the methylation code, and finally transform them into diverse functional signals are called m6A “readers” [14].

Recent studies have demonstrated that m6A modification is associated with various human diseases [15, 16]. However, there is no study to characterize m6A modification in patients with SLE. To investigate whether m6A modification plays a role in the genesis and development of SLE, the mRNA levels of *METTL3*, *MTEEL14*, *WTAP*, *FTO*, *ALKBH5*, and *YTHDF2* in peripheral blood were detected in SLE patients and analyzed for their correlation with clinical variables.

## 2. Methods

### 2.1. Patient Variables and Controls

A total of 51 patients that fulfilled the revised American College of Rheumatology criteria for SLE [17] were recruited from the First Affiliated Hospital of Nanchang University from 2018.10 to 2019.3. Among them, 40 patients were new-onset SLE that first-time diagnosis of SLE and no history of immunosuppressive drug or corticosteroid use before recruitment. Among all new-onset SLE patients, 7 patients were reexamined after 15 days of regular treatment by using glucocorticoids and immunosuppressive agents. The other 11 patients were revisiting SLE patients receiving treatment. Disease activity was assessed by the SLE disease activity index (SLEDAI) [18]. 38 healthy controls (CON) without a clinical diagnosis of any inflammatory or autoimmune diseases and without relation to patients of autoimmune disease were enrolled from the First Affiliated Hospital of Nanchang University. In addition, 51 patients fulfilled the revised ACR 2010 criteria for rheumatoid arthritis (RA) [19], 30 patients were infected with hepatitis B virus (HBV) and 27 patients with tuberculosis (TB) were recruited from the First Affiliated Hospital of Nanchang University. The demographic characteristics of the study population are shown in Table 1. The study had approval from the Ethics Committee of the First Affiliated Hospital of Nanchang University (052) and complied with the Helsinki Declaration. All participants provided signed informed consent before they entered this study.

### 2.2. Blood Sample Collection and Total RNA Isolation

The peripheral blood collection and total RNA isolation were performed as described previously [20].

### 2.3. QRT-PCR Analysis

Complementary DNA (cDNA) was obtained from purified total RNA samples by reverse transcription using a PrimeScript™ RT reagent kit (Takara Bio Inc., Japan). *METTL3*, *METTL14*, *WTAP*, *ALKBH5*, *FTO*, and *YTHDF2* transcripts were quantified with an ABI 7500 Real-time PCR System (Applied Biosystems; Thermo Fisher Scientific, Inc.) by using SYBR® Premix Ex Taq™ II (Takara Bio Inc., Japan). The sequences of amplification primers for *METTL3*, *METTL14*, *WTAP*, *ALKBH5*, *FTO*, *YTHDF2*, and GAPDH are listed in Table 2. The relative levels of transcripts were derived by the 2^-*Δ*Ct^ method [20, 21].

### 2.4. Blood Routine, Urinary Routine, Serum Inflammatory Indicators, and Autoantibody Determination

The items in blood routine and urinary routine were detected using Sysmex-x2100 (SYSMEX, Kobe, Japan) and SysmexUF-1000i (SYSMEX, Japan). Erythrocyte sedimentation rate (ESR) was determined by the BlessLBY-XC40B automatic ESR analyzer (Bless, Beijing, China) according to the manufacturer's instructions. The levels of C-reactive protein (CRP), immunoglobulin G (IgG), complement 3 (C3), and complement 4 (C4) in serum were determined by nephelometry methods using IMMAGE800 system (Beckman, CA, USA). Anti-dsDNA of IgG in serum was measured by commercially available enzyme-linked immunosorbent assay (ELISA) kits (Kexin, Shanghai, China). Antiextractable nuclear antigen (Anti-ENA) was determined using line immunoassay kits (Euroimmun, Luebeck, Germany) according to the manufacturer's instructions.

### 2.5. Statistical Analysis

Student's *t*-test or the Mann-Whitney *U*-test was used to compare the data according to the normality. The Spearman method was used for correlation analysis. Logistic regression analysis was used for evaluating the risk factor. All the data were analyzed by GraphPad Prism version 5.0 (GraphPad Software, San Diego, CA) and SPSS version 17.0 (SPSS Inc., Chicago, IL). *P* values < 0.05 were considered statistically significant.

## 3. Results

### 3.1. Differential Expression Screening of METTL3, METTL14, WTAP, ALKBH5, FTO, and YTHDF2 in Peripheral Blood of SLE Patients

**Figure 1:**
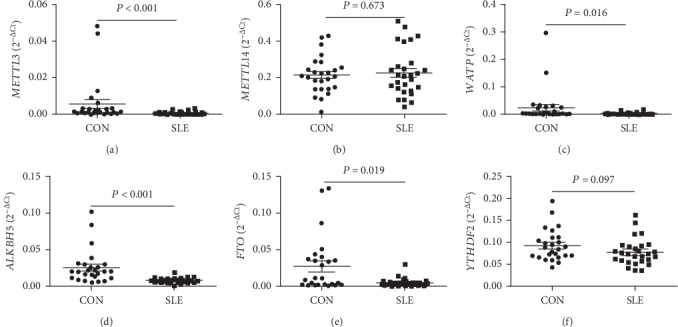
Differential expression screening of METTL3, METTL14, WTAP, ALKBH5, FTO, and YTHDF2 in peripheral blood of SLE patients. (a) The level of *METTL3* in peripheral blood was significantly decreased in SLE patients (*n* = 28) compared to healthy controls (CON, *n* = 26). (b) The level of *METTL14* showed no significant difference between SLE patients (*n* = 28) and CON (*n* = 26). (c) The level of *WTAP* in peripheral blood was significantly decreased in SLE patients (*n* = 28) compared to CON (*n* = 26). (d) The level of *ALKBH5* in peripheral blood was significantly decreased in SLE patients (*n* = 28) compared to CON (*n* = 26). (e) The level of *FTO* in peripheral blood was significantly decreased in SLE patients (*n* = 28) compared to CON (*n* = 26). (f) The level of *YTHDF2* trends to decline in SLE patients, but a significant difference was not reached.

To identify the expression levels of *METTL3*, *METTL14*, *WTAP*, *ALKBH5*, *FTO*, and *YTHDF2* in patients with SLE, the mRNA levels of these genes in peripheral blood were determined by qRT-PCR in SLE patients and HCs. Data showed that the mRNA levels of *METTL3*, *WTAP*, *ALKBH5*, and *FTO* in peripheral blood of SLE patients significantly decreased compared to HCs (all *P* < 0.050) (Figure 1). The mRNA level of *YTHDF2* trends to decrease in SLE patients, but a significant difference was not reached (*P* = 0.097) (Figure 1). The mRNA level of *METTL14* was unchanged (*P* = 0.673) (Figure 1).

### 3.2. Differential Expression Validation of METTL3, WTAP, ALKBH5, FTO, and YTHDF2

**Figure 2:**
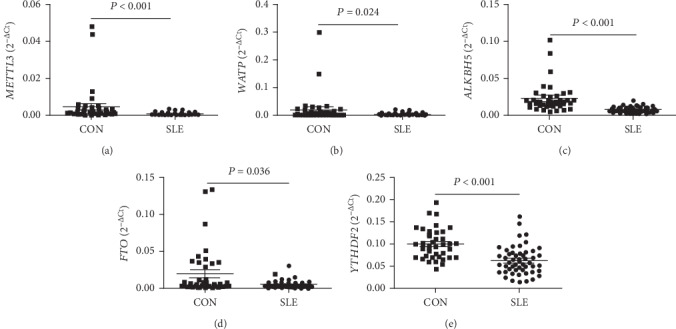
Differential expression validation of peripheral blood METTL3, WTAP, ALKBH5, FTO, and YTHDF2. (a) The level of *METTL3* in peripheral blood was significantly decreased in SLE patients (*n* = 51) compared to healthy controls (CON, *n* = 38). (b) The level of *WTAP* in peripheral blood was significantly decreased in SLE patients (*n* = 51) compared to CON (*n* = 38). (c) The level of *ALKBH5* in peripheral blood was significantly decreased in SLE patients (*n* = 51) compared to CON (*n* = 38). (d) The level of *FTO* in peripheral blood was significantly decreased in SLE patients (*n* = 51) compared to CON (*n* = 38). (e) The level of *YTHDF2* in peripheral blood was significantly decreased in SLE patients (*n* = 51) compared to CON (*n* = 38).

To further verify the differential expression of *METTL3*, *WTAP*, *ALKBH5*, *FTO*, and *YTHDF2* in peripheral blood in the screening stage, an independent cohort including 23 SLE patients and 12 HCs were recruited and detected for the mRNA levels of these genes in peripheral blood. The data from all subjects, including all 51 SLE patients and 38 HCs in screening stage and validation stage, demonstrated that the mRNA levels of *METTL3*, *WTAP*, *ALKBH5*, and *FTO* in peripheral blood were significantly lower in SLE patients than those in HCs (all *P* < 0.050) (Figure 2). Inconsistent with the screening data, the level of *YTHDF2* was also significantly decreased in SLE patients than that in HCs (*P* < 0.001) (Figure 2).

### 3.3. Correlations between the mRNA Levels of Peripheral Blood METTL3, WTAP, ALKBH5, FTO, and YTHDF2 and Autoantibodies in SLE Patients

**Figure 3:**
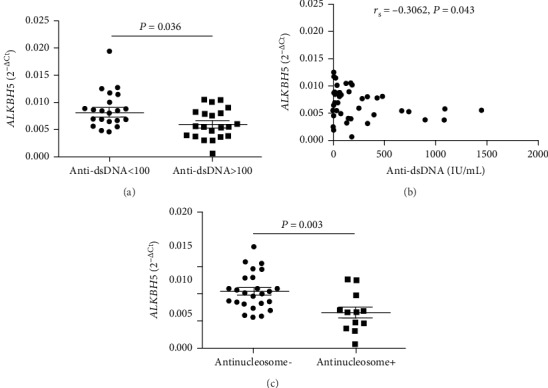
Correlations between the mRNA level of peripheral blood *ALKBH5* and autoantibodies in SLE patients. (a) The mRNA level of peripheral blood *ALKBH5* was significantly decreased in SLE patients with positive anti-double-stranded DNA (anti-dsDNA) compared to SLE patients with negative anti-dsDNA. (b) The mRNA level of peripheral blood *ALKBH5* in SLE patients negatively correlated with the level of anti-dsDNA. (c) The level of peripheral blood *ALKBH5* was significantly decreased in SLE patients with positive antinucleosome compared to SLE patients with negative antinucleosome.

Production of multiple autoantibodies such as anti-double-stranded DNA (anti-dsDNA) and antiextractable nuclear antigens (Anti-ENAs) is one of the important characteristics of SLE. In 51 SLE patients, 44 SLE patients were tested for anti-dsDNA and 37 SLE patients were tested for anti-ENAs. The correlations between the mRNA levels of peripheral blood *METTL3*, *WTAP*, *ALKBH5*, *FTO*, and *YTHDF2* and autoantibodies were investigated in these SLE patients. As shown in Figure 3(a), the level of peripheral blood *ALKBH5* was significantly decreased in patients with positive anti-dsDNA compared to patients with negative anti-dsDNA. Furthermore, the level of peripheral blood *ALKBH5* in SLE patients negatively correlated with the level of anti-dsDNA (*r*_s_ = −0.3062, *P* = 0.043) (Figure 3(b)). And, the level of peripheral blood *ALKBH5* was significantly decreased in patients with positive antinucleosome compared to patients with negative antinucleosome (*P* = 0.003) (Figure 3(c)). The level of peripheral blood *ALKBH5* did not correlate with other anti-ENAs (data not shown). No obvious correlation was observed between the levels of peripheral blood *METTL3*, *WTAP*, *FTO*, and *YTHDF2* and autoantibodies in SLE patients (data not shown).

### 3.4. Decreased mRNA Level of *ALKBH5* in Peripheral Blood Was a Risk Factor for SLE

**Figure 4:**
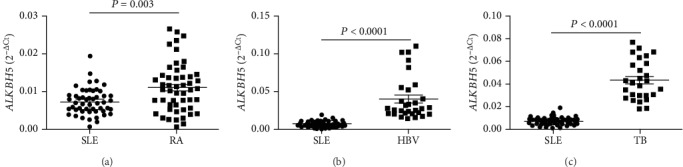
Validation of the abnormal expression of peripheral blood *ALKBH5* in SLE patients, rheumatoid arthritis (RA) patients, hepatitis B virus- (HBV-) infected patients, and tuberculosis (TB) patients. (a) The peripheral blood level of *ALKBH5* in SLE patients (*n* = 51) was significantly lower compared to RA patients (*n* = 51). (b) The peripheral blood level of *ALKBH5* in SLE patients (*n* = 51) was significantly lower compared to HBV-infected patients (*n* = 30). (c) The peripheral blood level of *ALKBH5* in SLE patients (*n* = 51) was significantly lower compared to TB patients (*n* = 27).

The aforementioned results demonstrated that the levels of peripheral blood *METTL3*, *WTAP*, *ALKBH5*, *FTO*, and *YTHDF2* in SLE patients were decreased and the level of *ALKBH5* in peripheral blood was associated with autoantibody production. Considering that the production of autoantibodies plays an important role in the pathogenesis of SLE, the “enter method” of multivariate logistic regression was used to explore whether the expression of peripheral blood *METTL3*, *WTAP*, *ALKBH5*, *FTO*, and *YTHDF2* was risk factors for SLE. As shown in Table 3, the equations about the levels of peripheral blood *METTL3*, *WTAP*, *ALKBH5*, *FTO*, and *YTHDF2* were obtained, *Y* = −326.128 X1(*METTL*3) − 74.188 (*WTAP*) − 414.645 X3(*ALKBH*5) − 15.042 X4(*FTO*) − 0.106 X2(*YTHDF*2) + 6.197. The results demonstrated that the decreased expression of *ALKBH5* in peripheral blood was a risk factor for SLE (*P* < 0.001), while other molecules are not (*P* > 0.050). Furthermore, the mRNA level of peripheral blood *ALKBH5* in other inflammatory disorders including RA, TB, and HBV-infected patients was detected and compared with that in SLE patients. Results showed that the mRNA level of peripheral blood *ALKBH5* in SLE patients was significantly lower compared to RA patients (*P* = 0.003) (Figure 4(a)), HBV-infected patients (*P* < 0.0001) (Figure 4(b)), and TB patients (*P* < 0.0001) (Figure 4(c)).

### 3.5. Correlations between the Levels of Peripheral Blood METTL3, WTAP, ALKBH5, FTO, and YTHDF2 and Clinical Variables in SLE Patients

**Figure 5:**
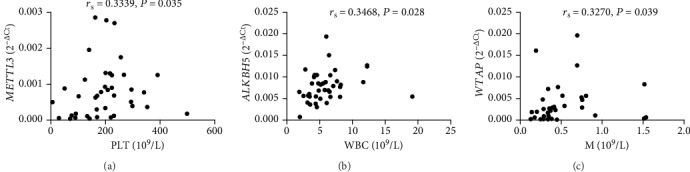
Correlations between peripheral blood *METTL3*, *ALKBH5* and *WTAP* and clinical variables in new-onset SLE patients. (a) The level of peripheral blood *METTL3* in new-onset SLE patients positively correlated with PLT (*n* = 40). (b) The level of peripheral blood *ALKBH5* in new-onset SLE patients positively correlated with WBC (*n* = 40). (c) The level of peripheral blood *WTAP* in new-onset SLE patients positively correlated with M (*n* = 40).

The aforementioned results demonstrate that the level of peripheral blood *ALKBH5* in SLE patients was negatively correlated with the level of anti-dsDNA. Anti-dsDNA was proved to be involved in the genesis of SLE and can be served as markers for evaluating ongoing disease activity in SLE and early relapse of SLE. And, the level of anti-dsDNA in SLE patients was found to negatively correlate with the level of C3 (*r* = −0.4244, *P* = 0.006) (Supplement Fig 1A) and C4 (*r* = −0.3905, *P* = 0.012) (Supplement Fig 1B) and positively correlate with SLEDAI (*r* = 0.3575, *P* = 0.024) (Supplement Fig 1C). Thus, the correlation test was performed to evaluate the correlations between the clinical variables of SLE and the levels of peripheral blood *METTL3*, *WTAP*, *ALKBH5*, *FTO*, and *YTHDF2.* Data showed that the mRNA levels of peripheral blood *METTL3*, *WTAP*, *ALKBH5*, *FTO*, and *YTHDF2* in all SLE patients (including all new-onset and revisiting SLE patients) did not correlate with SLEDAI, CRP, ESR, IgG, C3, C4, WBC, RBC, HGB, HCT, PLT, L, M, or N (data not shown). However, further analysis in the new-onset SLE patients showed that the mRNA level of peripheral blood *METTL3* positively correlated with PLT (*r*_s_ = 0.3339, *P* = 0.035) (Figure 5(a)), the mRNA level of peripheral blood *ALKBH5* positively correlated with WBC (*r*_s_ = 0.3468, *P* = 0.028) (Figure 5(b)), and the mRNA level of peripheral blood *WTAP* positively correlated with M (*r*_s_ = 0.3270, *P* = 0.039) (Figure 5(c)).

Next, the relationship between the levels of these m6A modification-related molecules and drug therapy of SLE was analyzed. However, we found no significant difference between new-onset SLE patients and revisiting SLE patients (data not shown). Subsequently, a 15-day follow-up evaluation in 7 new-onset SLE patients who received regular treatment was performed, but there was still no difference (data not shown).

**Figure 6:**
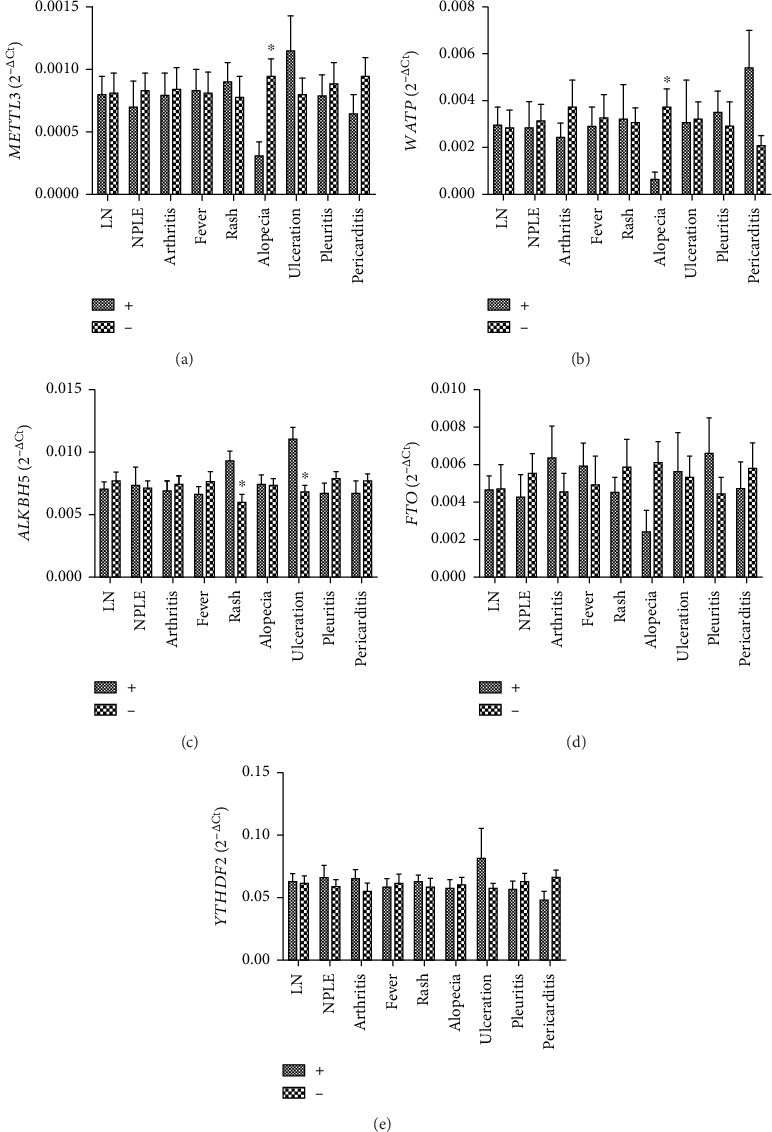
Correlations between peripheral blood *METTL3*, *WTAP*, *ALKBH5*, *FTO*, and *YTHDF2* and clinical symptoms of SLE. (a) The level of peripheral blood *METTL3* in SLE patients with alopecia was significantly decreased than that in SLE patients without alopecia. (b) The level of peripheral blood *WTAP* in SLE patients with alopecia was significantly decreased than that in SLE patients without alopecia. (c) The levels of peripheral blood *ALKBH5* in SLE patients with rash and ulceration were significantly increased than those in SLE patients without rash and ulceration, respectively. (d) No correlation was found between peripheral blood *FTO* and clinical symptoms of SLE. (e) No correlation was found between peripheral blood *YTHDF2* and clinical symptoms of SLE.

Moreover, the relationship between the clinical symptoms of SLE including LN, NPLE, arthritis, fever, rash, alopecia, ulceration, pleuritis, pericarditis, and the mRNA levels of these m6A modification-related molecules was analyzed. As shown in Figure 6, the mRNA levels of peripheral blood *METTL3* and *WTAP* in SLE patients with alopecia was significantly decreased than that in SLE patients without alopecia (all *P* < 0.050); the mRNA level of peripheral blood *ALKBH5* in SLE patients with rash and ulceration was significantly increased than that in SLE patients without rash and ulceration, respectively (all *P* < 0.050). No relationship was found between the levels of peripheral blood *FTO*, *YTHDF2*, and SLE symptoms (Figures 6(d) and 6(e)).

### 3.6. Correlations between the Levels of These m6A Modification-Related Molecules in SLE Patients

**Figure 7:**
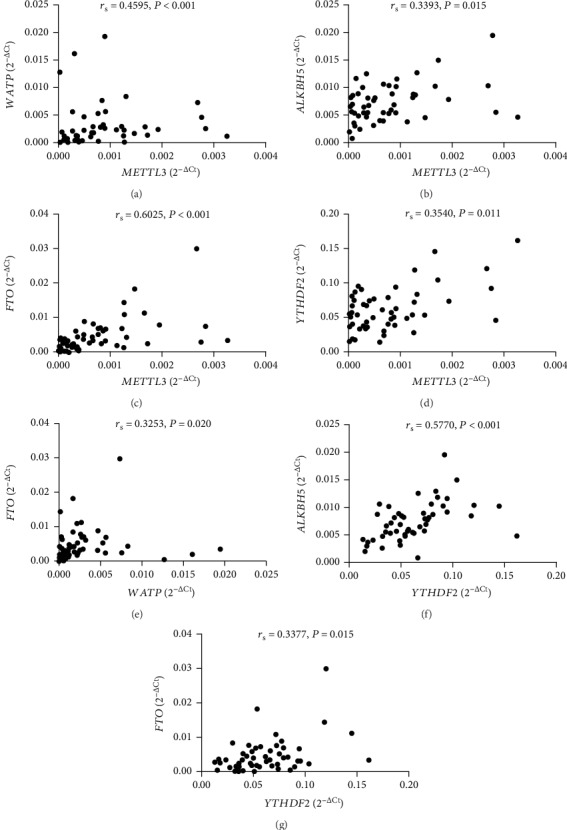
Interrelations between peripheral blood *METTL3*, *WTAP*, *ALKBH5*, *FTO*, and *YTHDF2* in SLE patients. (a) The level of peripheral blood *METTL3* positively correlated with *WTAP.* (b) The level of peripheral blood *METTL3* positively correlated with *ALKBH5.* (c) The level of peripheral blood *METTL3* positively correlated with *FTO*. (d) The level of peripheral blood *METTL3* positively correlated with *YTHDF2.* (e) The level of peripheral blood *WTAP* positively correlated with *FTO*. (f) The level of peripheral blood *YTHDF2* positively correlated with *ALKBH5.* (g) The level of peripheral blood *YTHDF2* positively correlated with *FTO*.

Considering that METTL3, WTAP, ALKBH5, FTO, and YTHDF2 are all key molecules in the process of m6A modification, we therefore want to know whether they are interrelated. As shown in Figure 5, the mRNA level of peripheral blood *METTL3* positively correlated with the mRNA levels of *WTAP* (*r*_s_ = 0.4595, *P* < 0.001) (Figure 7(a)), *ALKBH5* (*r*_s_ = 0.3393, *P* = 0.015) (Figure 7(b)), *FTO* (*r*_s_ = 0.6025, *P* < 0.001) (Figure 7(c)), and *YTHDF2* (*r*_s_ = 0.3540, *P* = 0.011) (Figure 7(d)); the mRNA level of peripheral blood *WTAP* positively correlated with the mRNA level of *FTO* (*r*_s_ = 0.3253, *P* = 0.020) (Figure 7(e)); and the mRNA level of peripheral blood *YTHDF2* positively correlated with the mRNA levels of *ALKBH5* (*r*_s_ = 0.5770, *P* < 0.001) (Figure 7(f)) and *FTO* (*r*_s_ = 0.3377, *P* = 0.015) (Figure 7(g)).

## 4. Discussion

Epigenetic alterations, including DNA methylation and histone modifications, have been reported to contribute to SLE progression [8, 22, 23]. Zhao et al. showed that RFX1 regulates CD70 and CD11a expression in lupus T cells by recruiting the histone methyltransferase SUV39H1, triggering autoimmune responses [24]. In recent years, the fields of m6A modification and epitranscriptomics have attracted much attention [25]. As an important form of posttranscriptional gene regulation, m6A modification was found to be involved in almost all aspects of RNA metabolism, which is widely involved in the T cell response to HIV infection [26], type I interferon production [27], and T cell differentiation and homeostasis [28]. Thus, it invites the speculation that m6A modification may be involved in the genesis and progression of SLE [13].

As the regulators of m6A methylation, METTL3, MTEEL14, WTAP, FTO, ALKBH5, and YTHDF2 play pivotal roles in the dynamic regulation of m6A modification. Nettersheim et al. showed that the mRNA levels of *METTL3*, *MTEEL14*, *WTAP*, and *ALKBH5* were significantly decreased in testicular germ cell tumors and the m6A modification in RNA of testicular germ cell tumors was controlled by *METTL3*, *ALKBH5*, and *YTHDF2* [29]. Tian et al. indicated that the mRNA expressions of *METTL3* and *FTO* during the osteogenic induction of BMSCs were increased [30]. In addition, Yang et al. demonstrated that the mRNA levels of *FTO*, *METTL3*, *METTL14*, and *WTAP* were increased in patients with type-2 diabetes [16]. These studies indicated m6A methylation regulators including m6A methylation writers, erasers, and readers which were changed synchronously. In this study, we also found that the mRNA levels of m6A methylation writers (*METTL3* and *WTAP*), erasers (*ALKBH5* and *FTO*), and reader *(YTHDF2)* were all decreased in peripheral blood from SLE patients. In accordance with the results of Yang et al. [16], our results manifested that the mRNA levels of peripheral blood m6A methylation writers positively correlated with those of erasers and reader, and the mRNA levels of peripheral blood m6A methylation erasers positively correlated with that of m6A methylation reader in SLE patients. Considering the fact that the collaboration among writers, erasers, and readers sets up the m6A threshold and perturbs that m6A threshold leads to uncontrolled expression/activity of virulence gene and results in the occurrence and development of diseases [31], our study suggested that the decreased *METTL3*, *WTAP*, *ALKBH5*, *FTO*, and *YTHDF2* may play an important role in the pathogenesis of SLE.

SLE is a systemic autoimmune disease characterized by elevated autoimmune antibodies, such as anti-dsDNA and antinucleosome. It is well known that anti-dsDNA and antinucleosome are pathogenic autoantibodies involved in organ damage of SLE [32–34]. In this study, we found that the level of peripheral blood *ALKBH5* was significantly decreased in SLE patients compared to HCs, RA patients, TB patients, and HBV-infected patients. Furthermore, the level of peripheral blood *ALKBH5* significantly decreased in SLE patients with positive anti-dsDNA or antinucleosome compared to SLE patients with negative anti-dsDNA or antinucleosome. And, the level of peripheral blood *ALKBH5* in SLE patients was found to be negatively correlated with the level of anti-dsDNA. In addition, the level of peripheral blood *ALKBH5* in SLE patients was associated with clinical symptoms of SLE such as rash, ulceration, and leukopenia. Logistic regression analysis revealed that decreased mRNA level of *ALKBH5* in peripheral blood was a risk factor for SLE.

Considering that anti-dsDNA and antinucleosome are involved in the pathogenesis of SLE and are specific diagnostic biomarkers of SLE, our results suggested that the expression of peripheral blood *ALKBH5* may be involved in the pathogenesis of SLE and may also serve as a potential biomarker for SLE diagnosis and severity evaluation.

In conclusion, it is the first study to our knowledge that explored the mRNA levels of peripheral blood *METTL3*, *METTL14*, *WTAP*, *ALKBH5*, *FTO*, and *YTHDF2* in SLE patients. In this study, we found that the mRNA levels of *METTL3*, *WTAP*, *ALKBH5*, *FTO*, and *YTHDF2* in peripheral blood from SLE patients were significantly decreased. Additionally, this study established the correlations between the mRNA level of *ALKBH5* and the production of autoantibodies, clinical features, which might improve our understanding of the role of *ALKBH5* in SLE.

## Figures and Tables

**Table 1:** Clinical characteristics of SLE patients, RA patients, HBV-infected patients, TB patients, and CON.

Categories	SLE patients	CON	RA patients	HBV	TB patients
*n*	51	38	51	30	27
Females (%)	92.16	78.95	82.35	93.33	100
Age (years)	39.30 ± 14.37	39.71 ± 13.48	50.67 ± 12.67	35.93 ± 8.04	33.46 ± 12.05
SLEDAI score	13.09 ± 7.75				
Anti-dsDNA (IU/mL)	230.40 ± 342.73				
Anti-ENA					
Anti-Sm (%)	29.73				
Anti-Ro52 (%)	70.27				
Anti-nRNP/Sm (%)	59.46				
Anti-RIB-P (%)	24.32				
Antinucleosome (%)	32.43				
Anti-SSA (%)	64.86				
Anti-SSB (%)	18.92				
C3 (g/L)	0.59 ± 0.27				
C4 (g/L)	0.14 ± 0.11				
IgG (g/L)	18.66 ± 6.00				
ESR (mm/h)	62.07 ± 31.13				
CRP (mg/L)	18.63 ± 35.25				
WBC (10^9^/L)	6.43 ± 3.90	5.84 ± 0.96			
RBC (10^12^/L)	3.80 ± 0.67^∗^	4.59 ± 0.39			
HGB (g/L)	124.41 ± 76.06^∗^	138.58 ± 11.50			
HCT (L/L)	0.34 ± 0.06^∗^	0.41 ± 0.03			
PLT (10^9^/L)	195.83 ± 94.01^∗^	243.00 ± 46.32			
Lymphocytes (10^9^/L)	1.39 ± 0.62^∗^	1.86 ± 0.30			
Lymphocytes (%)	25.08 ± 10.26^∗^	32.51 ± 6.30			
Monocytes (10^9^/L)	0.51 ± 0.34^∗^	0.36 ± 0.09			
Monocytes (%)	8.09 ± 2.66^∗^	6.14 ± 1.43			
Neutrophils (10^9^/L)	4.45 ± 3.41	3.52 ± 0.82			
Neutrophils (%)	65.49 ± 11.10^∗^	59.49 ± 6.00			
Clinical features					
Fever (%)	44.44				
Rash (%)	36.11				
Alopecia (%)	17.14				
Arthritis (%)	47.22				
NPLE (%)	11.11				
Ulceration (%)	11.43				
Pleuritis (%)	45.71				
Pericarditis (%)	34.29				
LN (%)	54.90				

^∗^ *P* < 0.05 SLE compared to CON. Anti-dsDNA: anti-double-stranded DNA; Anti-ENA: antiextractable nuclear antigen; Anti-nRNP/Sm: antinuclear ribonucleoprotein/Smith antibody; Anti-RIB-P: anti-ribosomal P-protein antibody; Anti-Sm: anti-Smith antibody; Anti-SSA: anti-Sjögren syndrome A antigen antibody; Anti-SS-B: anti-Sjögren syndrome B antigen antibody; HBV: hepatitis B virus (HBV); C3: complement 3; C4: complement 4; HC: healthy controls; CRP: C-reactive protein; ESR: erythrocyte sedimentation rate; HCT: hematocrit; HGB: hemoglobin; LN: lupus nephritis; IgG: immunoglobulin G; L: lymphocyte count; L%: lymphocyte percentage; M: monocyte count; M%: monocyte percentage; N: neutrophil count; N%: neutrophil percentage; NPLE: neuropathic lupus erythematosus; PLT: platelet count; RBC: red blood cell count; RA: rheumatoid arthritis; SLE: systemic lupus erythematosus; SLEDAI: SLE disease activity index; TB: tuberculosis; WBC: white blood cell count.

**Table 2:** The amplification primers sequences.

Gene name	Sequence (5′-3′)
*METTL3*	F: AAGCTGCACTTCAGACGAAT
	R: GGAATCACCTCCGACACTC
*METTL14*	F: AGAAACTTGCAGGGCTTCCT
	R: TCTTCTTCATATGGCAAATTTTCTT
*WTAP*	F: GGCGAAGTGTCGAATGCT
	R: CCAACTGCTGGCGTGTCT
*ALKBH5*	F: CCCGAGGGCTTCGTCAACA
	R: CGACACCCGAATAGGCTTGA
*FTO*	F: TGGGTTCATCCTACAACGG
	R: CCTCTTCAGGGCCTTCAC
*YTHDF2*	F: GGCAGCACTGAAGTTGGG
	R: CTATTGGAAGCCACGATGTTA
GAPDH	F: TGCACCACCAACTGCTTAGC
	R: GGCATGGACTGTGGTCATGAG

*METTL3*: methyltransferase-like 3; *METTL14*: methyltransferase-like 14; *WTAP*: Wilms tumor 1-associating protein; *ALKBH5*: a-ketoglutarate-dependent dioxygenase alkB homolog 5; *FTO*: fat mass and obesity-associated protein; *YTHDF2*: YT521-B homology domains 2.

**Table 3:** The expression of *METTL3*, *WTAP*, *ALKBH5*, *FTO*, and *YTHDF2* in equation.

	B	S.E	Wald	df	*P*	Exp (B)
*METTL3*	-326.128	272.636	1.431	1	0.232	0.000
*WTAP*	-74.188	66.854	1.231	1	0.267	0.000
*ALKBH5*	-414.645	108.988	14.474	1	0.000	0.000
*FTO*	-15.042	58.027	0.067	1	0.795	0.000
*YTHDF2*	-0.106	14.726	0.000	1	0.994	0.899
Constant	6.197	1.408	19.373	1	0.000	491.119

*METTL3*: methyltransferase-like 3; *WTAP*: Wilms tumor 1-associating protein; *ALKBH5*: a-ketoglutarate-dependent dioxygenase alkB homolog 5; *FTO*: fat mass and obesity-associated protein; *YTHDF2*: YT521-B homology domains 2.

## Data Availability

The data used to support the findings of this study are available from the corresponding author upon request.

## Supplementary Materials

Supplementary Materials Supplement Figure 1: correlations between anti-double-stranded DNA (anti-dsDNA) and complement 3 (C3), complement 4 (C4), and systemic lupus erythematosus disease activity index (SLEDAI) in systemic lupus erythematosus (SLE) patients. (a) The level of anti-dsDNA negatively correlated with that of C3. (b) The level of anti-dsDNA negatively correlated with that of C4. (c) The level of anti-dsDNA positively correlated with SLEDAI.

## References

[1] Frieri M. (2013). Mechanisms of disease for the clinician: systemic lupus erythematosus. *Annals of Allergy, Asthma & Immunology*.

[2] D'Cruz D. P., Khamashta M. A., Hughes G. R. (2007). Systemic lupus erythematosus. *Lancet*.

[3] Jenks S. A., Sanz I. (2009). Altered B cell receptor signaling in human systemic lupus erythematosus. *Autoimmunity Reviews*.

[4] Crispin J. C., Kyttaris V. C., Terhorst C., Tsokos G. C. (2010). T cells as therapeutic targets in SLE. *Nature Reviews Rheumatology*.

[5] Luo Q., Huang Z., Ye J. (2016). PD-L1-expressing neutrophils as a novel indicator to assess disease activity and severity of systemic lupus erythematosus. *Arthritis Research & Therapy*.

[6] Tsokos G. C. (2011). Systemic lupus erythematosus. *The New England Journal of Medicine*.

[7] La Paglia G. M. C., Leone M. C., Lepri G. (2017). One year in review 2017: systemic lupus erythematosus. *Clinical and Experimental Rheumatology*.

[8] Zhu H., Mi W., Luo H. (2016). Whole-genome transcription and DNA methylation analysis of peripheral blood mononuclear cells identified aberrant gene regulation pathways in systemic lupus erythematosus. *Arthritis Research & Therapy*.

[9] Wang X., Zhang C., Wu Z., Chen Y., Shi W. (2018). CircIBTK inhibits DNA demethylation and activation of AKT signaling pathway via miR-29b in peripheral blood mononuclear cells in systemic lupus erythematosus. *Arthritis Research & Therapy*.

[10] Wang X., Lu Z., Gomez A. (2014). *N*
^6^-methyladenosine-dependent regulation of messenger RNA stability. *Nature*.

[11] Pinello N., Sun S., Wong J. J. (2018). Aberrant expression of enzymes regulating m6A mRNA methylation: implication in cancer. *Cancer Biology & Medicine*.

[12] Tuncel G., Kalkan R. (2019). Importance of m N^6^-methyladenosine (m^6^A) RNA modification in cancer. *Medical Oncology*.

[13] Li L. J., Fan Y. G., Leng R. X., Pan H. F., Ye D. Q. (2018). Potential link between m^6^A modification and systemic lupus erythematosus. *Molecular Immunology*.

[14] Maity A., Das B. (2016). N6-methyladenosine modification in mRNA: machinery, function and implications for health and diseases. *The FEBS Journal*.

[15] Yang S., Wei J., Cui Y. H. (2019). m^6^A mRNA demethylase FTO regulates melanoma tumorigenicity and response to anti-PD-1 blockade. *Nature Communications*.

[16] Yang Y., Shen F., Huang W. (2019). Glucose is involved in the dynamic regulation of m6A in patients with type 2 diabetes. *The Journal of Clinical Endocrinology and Metabolism*.

[17] Tan E. M., Cohen A. S., Fries J. F. (1982). The 1982 revised criteria for the classification of systemic lupus erythematosus. *Arthritis and Rheumatism*.

[18] Bombardier C., Gladman D. D., Urowitz M. B. (1992). Derivation of the SLEDAI. A disease activity index for lupus patients. *Arthritis and Rheumatism*.

[19] Aletaha D., Neogi T., Silman A. J. (2010). 2010 rheumatoid arthritis classification criteria: an American College of Rheumatology/European League Against Rheumatism collaborative initiative. *Arthritis and Rheumatism*.

[20] Luo Q., Zhang L., Li X. (2018). Identification of circular RNAs hsa_circ_0044235 in peripheral blood as novel biomarkers for rheumatoid arthritis. *Clinical and Experimental Immunology*.

[21] Zhang F., Wu L., Qian J. (2016). Identification of the long noncoding RNA NEAT1 as a novel inflammatory regulator acting through MAPK pathway in human lupus. *Journal of Autoimmunity*.

[22] Wu H., Zhao M., Tan L., Lu Q. (2016). The key culprit in the pathogenesis of systemic lupus erythematosus: aberrant DNA methylation. *Autoimmunity Reviews*.

[23] Renaudineau Y., Youinou P. (2011). Epigenetics and autoimmunity, with special emphasis on methylation. *The Keio Journal of Medicine*.

[24] Zhao M., Wu X., Zhang Q. (2010). RFX1 regulates CD70 and CD11a expression in lupus T cells by recruiting the histone methyltransferase SUV39H1. *Arthritis Research & Therapy*.

[25] Yang Y., Huang W., Huang J. T. (2016). Increased *N*^6^-methyladenosine in Human Sperm RNA as a Risk Factor for Asthenozoospermia. *Scientific Reports*.

[26] Lichinchi G., Gao S., Saletore Y. (2016). Dynamics of the human and viral m^6^A RNA methylomes during HIV-1 infection of T cells. *Nature Microbiology*.

[27] Zheng Q., Hou J., Zhou Y., Li Z., Cao X. (2017). The RNA helicase DDX46 inhibits innate immunity by entrapping m^6^A-demethylated antiviral transcripts in the nucleus. *Nature Immunology*.

[28] Li H. B., Tong J., Zhu S. (2017). m^6^A mRNA methylation controls T cell homeostasis by targeting the IL-7/STAT5/SOCS pathways. *Nature*.

[29] Nettersheim D., Berger D., Jostes S., Kristiansen G., Lochnit G., Schorle H. (2019). N6-Methyladenosine detected inRNAof testicular germ cell tumors is controlled byMETTL3,ALKBH5,YTHDC1/F1/F2, andHNRNPCas writers, erasers, and readers. *Andrology*.

[30] Tian C., Huang Y., Li Q., Feng Z., Xu Q. (2019). Mettl3 regulates osteogenic differentiation and alternative splicing of Vegfa in bone marrow mesenchymal stem cells. *International Journal of Molecular Sciences*.

[31] Panneerdoss S., Eedunuri V. K., Yadav P. (2018). Cross-talk among writers, readers, and erasers of m6A regulates cancer growth and progression. *Science Advances*.

[32] Yung S., Chan T. M. (2015). Mechanisms of kidney injury in lupus nephritis—the role of anti-dsDNA antibodies. *Frontiers in Immunology*.

[33] Gómez-Puerta J. A., Burlingame R. W., Cervera R. (2008). Anti-chromatin (anti-nucleosome) antibodies: diagnostic and clinical value. *Autoimmunity Reviews*.

[34] Manson J. J., Ma A., Rogers P. (2009). Relationship between anti-dsDNA, anti-nucleosome and anti-alpha-actinin antibodies and markers of renal disease in patients with lupus nephritis: a prospective longitudinal study. *Arthritis Research & Therapy*.

