# Supplementary figures and images for: Decreased Peripheral Blood ALKBH5 Correlates with Markers of Autoimmune Response in Systemic Lupus Erythematosus
Source: Dis Markers. 2020 Jun 25;2020:8193895. doi: 10.1155/2020/8193895 (PMC7334764; doi:10.1155/2020/8193895)

Supplement figure 1


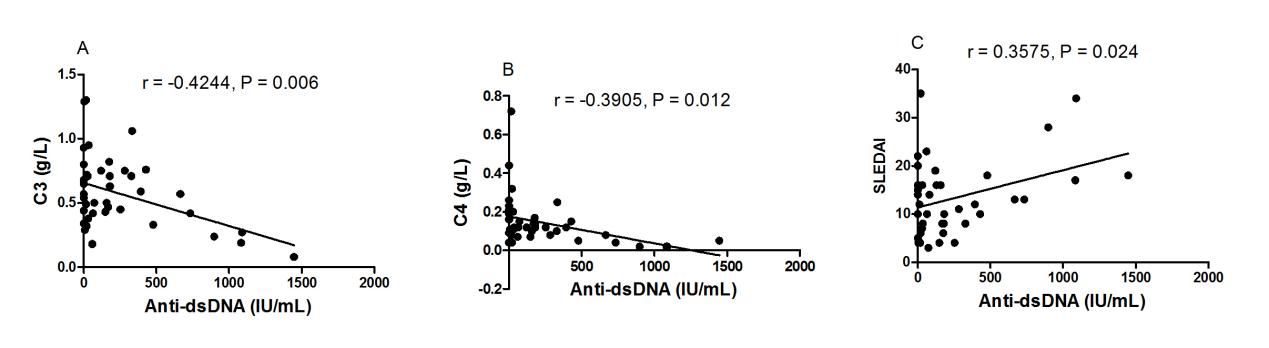

Supplement: Supplementary Materials — Supplement Figure 1: correlations between anti-double-stranded DNA (anti-dsDNA) and complement 3 (C3), complement 4 (C4), and systemic lupus erythematosus disease activity index (SLEDAI) in systemic lupus erythematosus (SLE) patients. (a) The level of anti-dsDNA negatively correlated with that of C3. (b) The level of anti-dsDNA negatively correlated with that of C4. (c) The level of anti-dsDNA positively correlated with SLEDAI. [file 8193895.f1.docx]
